# The Historical Relations of Medicine and Surgery to the End of the Sixteenth Century

**Published:** 1905-09

**Authors:** 


### Reviews of Books.

**The Historical Relations of Medicine and Surgery to the End of the Sixteenth Century.** By T. Clifford Allbutt, M.A., M.D. Pp. xvi., 125. London: Macmillan & Co. Limited. 1905.

This little book consists of an address delivered at the St. Louis Congress in 1904. It may be read with profit by all medical practitioners, and cannot fail to excite and hold the interest of its readers. It is enough to say to those who are already conversant with the author's writings that it is characterised by wide erudition and a marked historical sense, and written in the racy and incisive style which we expect from him. The book is practically a compendium of the history of medicine, and without being unduly condensed, and in consequence dry, it is yet remarkable for the amount of information which is contained within the compass of about a hundred and twenty pages. The theme is sufficiently indicated by the title, and is one of very present interest; for, as Prof. Allbutt shows, we are returning in practice, and that merely by the natural force of events, to the union of medical and surgical work in the same hands, in spite of the fact that such unity in practice has never been formally recognised by the colleges, whose existence and names perpetuate a distinction which he shows was purely artificial in origin and has been mischievous in result. For he has no difficulty in proving that in Greek and Roman times medicine and surgery were practised by the same man, and each gained from the association. Whether he is correct in further looking upon surgery as the active, living and inspiring part of medical science is perhaps more open to question. It was in the Middle Ages that the divorce came, arising from the mistaken notions of dignity that then prevailed and led to all manual work being regarded as degrading; and we should suggest partly also from the tendency for purposes of mutual support and protection to associate all workers at one art or craft into a guild or close corporation, and the guild once formed, there was the natural result that appears inherent in all trade associations—to make rules restricting the members of each guild to certain activities, in order to prevent them from infringing upon the similarly restricted work of other guilds.

Prof. Allbutt shows clearly the evils which arose from this unnatural division of medicine and surgery, especially to the detriment of the former, which was cut off from its experimental side. Perhaps the absurd notion that the practice of medicine is essentially superior to that of surgery has already died a natural death. If there be any that still cherish such an idea they will do well to study this work. Certainly surgery has in these days taken an ample revenge for the slight so long cast upon it by medicine by taking from the physicians an ever-increasing proportion of their work. Prof. Allbutt points out the mischief of such artificial constraints upon a man's natural bent in his work, and shows how mainly by the growth of specialism the old divorce between medicine and surgery has been perforce done away with. We may conclude, however, with a word of comfort to those who may fear that the craft and calling of a physician is in danger of extinction. The author does not propose to incontinently do away with physicians. The varied nature of medical work and of the mental acquirements needed for it make the division of labour into medical and surgical practice one which, not crystallised by artificial restraints, but left to its own free development, is natural and will endure. We strongly recommend this book to the perusal of our readers as one which is certain to interest them. Mention should be made of the good index.

